# Stressful life events and depression and fatigue in people with multiple sclerosis: a cross-sectional analysis of an international cohort

**DOI:** 10.1007/s13760-023-02390-z

**Published:** 2023-09-28

**Authors:** Jeanette C. Reece, Sandra L. Neate, Rebekah A. Davenport, Elasma Milanzi, Nupur Nag, William Bevens, Maggie Yu, George A. Jelinek, Steve Simpson-Yap

**Affiliations:** 1 Neuroepidemiology Unit, Centre for Epidemiology and Biostatistics, Melbourne School of Population and Global Health, Level 3, 207 Bouverie St, The University of Melbourne, VIC 3010 Australia; 2 Mood, Anxiety and Eating Disorders Laboratory, School of Psychological Sciences, The University of Melbourne, Australia; 3 Centre for Epidemiology and Biostatistics, Melbourne School of Population and Global Health, The University of Melbourne, Australia; 4 Australasian Kidney Trials Network, University of Queensland, Brisbane, Australia; 5 Menzies Institute for Medical Research, University of Tasmania, Hobart, Australia

**Keywords:** Multiple sclerosis, Stressful life event, Fatigue, Depressive symptoms

## Abstract

**Background:**

Relationships between stressful life events (SLEs) and health outcomes in people living with multiple sclerosis (plwMS), beyond relapse, are not well-established. We examined associations between SLEs and fatigue and symptoms of depression in plwMS.

**Methods:**

948 participants were queried whether they had experienced any of the 16 SLEs (Holmes–Rahe Social Readjustment Rating Scale) in the preceding 12 months. SLEs were summated to estimate SLE number and SLE load (weighted for the degree of associated stress). Cross-sectional associations between SLE (number, load and individual) and fatigue, and depressive symptoms were examined using log-binomial or log-multinomial regression adjusted for age, sex, relapse symptoms, education, MS type at baseline, disability, fatigue, comorbidity, depression, and antidepressant/antifatigue medications, as appropriate. Sub-analyses restricted to SLEs with a negative emotional impact were performed.

**Results:**

Median SLE number and load were 2 (IQR 1–2) and 57 (IQR 28–97), respectively. SLE number and load were not associated with a higher prevalence of fatigue, or depressive symptoms, even when restricting analyses to SLEs with a perceived negative emotional impact. A new relationship or family member with a negative impact was associated with a threefold and 2.5-fold higher prevalence of depressive symptoms, respectively. Serious illness was associated with a 28% higher prevalence of depressive symptoms.

**Conclusion:**

Psychological support for SLEs, and/or intervention targeted to SLE appraisal, may be beneficial in mitigating the adverse effects of SLEs with a perceived negative emotional impact on depressive symptoms in plwMS. Potential associations between serious illness and increased prevalence of depressive symptoms may warrant further investigation.

**Supplementary Information:**

The online version contains supplementary material available at 10.1007/s13760-023-02390-z.

## Introduction

The prevalence of stress in people living with multiple sclerosis (plwMS) is higher than the general population [1], with elevated levels of stress reported in ~ 48% of people with relapsing–remitting MS (RRMS) [2]. Psychological stress represents a route by which neuroinflammatory effects may manifest and potentially impact health outcomes in plwMS [3, 4]. However, the role of stress, or stressful life events (SLEs) in the course of MS, is not well-understood.

Meta-analysis of 14 studies found SLEs were moderately associated with MS exacerbation [weighted average size effect of 0.53 (95% CI 0.40–0.65)] [5], consistent with more recent studies [6, 7]. Other studies found stress was not related to relapse [8, 9], with an Israeli study reporting potentially protective effects of highly stressful war-time events on MS exacerbation [10]. However, inconsistencies across studies may be attributable to differences in study design, methods used to measure stress or relapse, or sample sizes. Therefore, studies to clarify associations between stress, particularly SLEs, and a range of health outcomes may help inform psychological interventions and clinical management.

Studies have also found associations between stress and reduced mental health outcomes, patient-reported health outcomes and quality of life (QoL) in plwMS. Higher perceived stress was associated with overall lower QoL, self-efficacy, and wellbeing in an Australian 2-year prospective study of 1287 plwMS [11]. Similarly, higher perceived stress levels were associated with lower physical and psychological QoL domains in a Polish correlational study of 100 plwMS [12]. A 2-year longitudinal Australian study reported a link between life-event stressors and predictors of fatigue in plwMS, but not depression [13]. As the prevalence of fatigue and depression in plwMS is high, but under-recognised in clinical care [14], studies examining depression and fatigue in relation to psychological stress may help clarify these associations and advise appropriate clinical care.

We examined the cross-sectional associations between SLE number, SLE load, and 16 individual SLEs with depressive symptoms and clinically significant fatigue using data from the prospective Health Outcomes and Lifestyle In a Sample of People with Multiple Sclerosis (HOLISM) study. Examining the link between SLEs and health outcomes in plwMS may help improve clinical management, especially those experiencing SLEs.

## Methods

### Participant characteristics

Data were analysed from HOLISM, a prospective cohort study comprising an international sample of plwMS, as described [15]. Inclusion criteria included participants ≥ 18 years at baseline and self-reported physician diagnosis of MS [16].

### Standard protocol approvals, registrations, and patient consents

The Strengthening the Reporting of Observational Studies in Epidemiology (STROBE) guidelines for cross-sectional studies were followed. Study approval was obtained by The University of Melbourne Human Research Ethics Committee (#1545102). All participants provided informed written consent.

### Measurements

HOLISM participants completed self-reported surveys related to sociodemographic and clinical characteristics, and prescription antidepressant and antifatigue medication use at baseline, 2.5-, 5- and 7.5-year follow-up. The present study analysed self-reported participant data at baseline and 7.5 years and SLE data at 7.5 years.

### Stressful life events (SLEs)

Participants were queried whether they had experienced 16 selected SLEs from the Holmes and Rahe Social Readjustment Rating Scale in the preceding 12 months (Supplementary Table 1) [17]. SLEs were chosen based on a previous study examining associations between SLEs and MS onset [18]. SLEs were summated to estimate ‘SLE number’. ‘SLE load’ was evaluated by summating weighted SLEs, with the death of a spouse or first-degree relative weighted “100”, and police encounter/court appearance considered less significant, and weighted “15” [19]. SLE number was evaluated as a continuous and categorised term (0, 1, or ≥ 2). SLE load was evaluated as a continuous term and categorised into quartiles.

Participants were asked to specify the perceived emotional impact of the SLE (negative vs positive), given not all SLEs have a negative impact [18]. The impact of individual SLEs was categorised as negative (− 5 to − 1), with − 5 representing the most negative impact, neutral (0), or positive (+ 1–+ 5), with + 5 representing the most positive impact.

### Outcome measures

Outcomes queried in the preceding 12 months included depressive symptoms and clinically significant fatigue. Symptoms of depression were assessed using the 9-item Patient Health Questionnaire-9 (PHQ-9), using > 9 to indicate major depressive symptoms which has 95% sensitivity and 85.9% specificity in plwMS [20]. Fatigue was assessed using the 9-item Fatigue Severity Score (FSS), with clinically significant fatigue defined as mean FSS > 5 [21]. Participants were also given a definition of relapses (the development of ≥ 1 new MS symptoms or worsening of existing symptoms lasting > 48 h, with changes in symptoms not due to extraneous conditions such as heat/illness, i.e., respiratory or urinary tract infections) and asked whether they were experiencing ongoing symptoms due to relapse in the preceding 30 days.

### Statistical analysis

Cross-sectional analysis was performed using data from the 7.5-year follow-up. Characteristics of SLE number (as a continuous variable and categorised as 0, 1, and ≥ 2, with 0 reference) and SLE load (as a continuous variable and categorised into quartiles as previously described [18]) were assessed using log-multinomial regression as univariable and multivariable analyses. Analyses were adjusted for age, sex, education, MS type at baseline, disability using the patient-reported outcome measure (PROM), Patient-reported MS Severity Score (P-MSSS), and fatigue, and antidepressant use.

SLEs associated with fatigue and depressive symptoms were assessed using log-binomial or log-multinomial regression, as appropriate. All analyses were routinely adjusted for experiencing ongoing symptoms due to a recent patient-reported relapse (model 1 adjustment), with participants asked whether they had symptoms due to a clinically confirmed relapse together with being provided with a comprehensive relapse definition, as in a prior HOLISM study [22]. Fatigue models were further adjusted for age, sex, P-MSSS, baseline treated comorbidity number, and antifatigue use (model 2 adjustment), then further adjusted for symptoms of depression to examine the effect of depressive symptoms on the SLE-fatigue association (model 3 adjustment). Depression models were further adjusted for age, sex, P-MSSS, and prescription antidepressant medication use (model 2 adjustment), and then further adjusted for clinically significant fatigue to examine the effect of fatigue on the SLE-depression association (model 3 adjustment). Results are presented as prevalence ratios (PRs) and 95% confidence intervals (CIs) [23]. Analyses were conducted using STATA SE/16.1 (StataCorp, USA).

Power calculations indicated analyses were sufficiently powered for examining associations between ≥ 2 SLEs and fatigue and symptoms of depression (Supplementary Table 2). There was sufficient power to measure an association between serious illness and fatigue, and symptoms of depression.

## Results

The 7.5-year follow-up cohort comprised 948 participants; mean age was 52.8 years, 80.2% were female and 72.5% were diagnosed with RRMS at baseline (Table 1). In all, 41.0% had clinically significant fatigue and 22.5% had depressive symptoms. Median time since MS onset was 18.1 years [interquartile range (IQR), 13.0–26.1] and median P-MSSS was 1.1 (IQR 0.3–3.9). Median SLE number and load were 2 (IQR, 0–9) and 57 (IQR, 28–97), respectively.

**Table 1:** Cohort characteristics at the 7.5-year review and MS type at baseline (*n* = 948)

Characteristic	*n* (%)
Sex	
Male	188 (19.8%)
Female	760 (80.2%)
MS type (baseline)	
RRMS	679 (72.5%)
SPMS	80 (8.5%)
PPMS	57 (6.1%)
PRMS	10 (1.1%)
Unsure (Missing)	111 (11.9%) (11 (1.2%))
Clinically significant fatigue	
No	506 (59.0%)
Yes (Missing)	352 (41.0%) (90 (9.5%))
Depressive symptoms (PHQ-9)	
No	664 (77.5%)
Yes (Missing)	193 (22.5%) (91 (9.6%))
Baseline treated comorbidity number	
0	735 (77.5%)
1	100 (10.6%)
≥ 2	113 (11.9%)
Immunomodulatory medication	
No	596 (62.9%)
Yes	352 (37.1%)
Prescription antidepressant medication	
No	747 (78.8%)
Yes	201 (21.2%)
Prescription antifatigue medication	
No	866 (91.4%)
Yes	82 (8.7%)
	Mean (SD; range)
Age	52.8 (10.2; 25.1–86.6)
	Median (IQR)
P-MSSS	1.1 (0.3–3.9)
Duration since MS onset, years	18.1 (13.0–26.1)
SLE number	2 (1–2)
SLE number (negative impact only)	1 (0–2)
SLE load	57 (28–97)
SLE load (negative impact only)	46 (0–83)

IQR, interquartile range; PHQ-9, Patient Health Questionnaire-9; P-MSSS, Patient-reported MS Severity Score; PPMS, primary progressive MS; PRMS, progressive relapsing MS; RRMS, relapsing–remitting MS; SLE, stressful life events; SPMS, secondary progressive MS

Due to the high attrition in the analysis sample at the 7.5-year follow-up (*n* = 948) compared with baseline (*n* = 2466), we examined the baseline characteristics of the analysis sample and those lost to follow-up (Supplementary Table 3). Compared to those lost to follow-up, the analysis sample had significantly fewer females, more people with RRMS at baseline and on immunomodulatory medication and fewer people with progressive MS, fatigue, symptoms of depression and ≥ 2 comorbidities.

### Characteristics of SLE number

SLE number was higher (≥ 2) among participants with moderate disability, clinically significant fatigue, and taking antidepressant medication. Other SLE characteristics are shown in Supplementary Table 4.

### SLEs and depressive symptoms

SLE number and load were associated with a 14% and 18% higher prevalence of depressive symptoms, respectively, after adjusting for symptoms due to recent relapse (Table 2; model 1 adjustment). On restricting analyses to SLEs with a negative impact, both SLE number and load were associated with a 20% higher prevalence of depressive symptoms, with an increasing trend in the prevalence with increasing SLEs. Associations were similar following model 2 adjustment for age, sex, baseline MS type, disability, and antidepressant medication. However, further adjustment for fatigue (model 3 adjustment) found associations between SLE number and load and depressive symptoms were no longer significant, indicating associations between SLE number and load and depression following model 2 adjustment may be attributable to SLEs acting via fatigue.

**Table 2:** SLE characteristics of depressive symptoms (PHQ-9)

SLE measure	*n*/*N* (row %)	aPR (95% CI)^a^	aPR (95% CI)^b^	aPR (95% CI)^c^
SLE number				
Continuous		**1.14 (1.05, 1.24)** ***p* = *0.002***	**1.09 (1.01, 1.17)** ***p* = *0.033***	1.04 (0.96, 1.13) *p* = *0.31*
0	15/77 (19.5%)	1.00 [Reference]	1.00 [Reference]	1.00 [Reference]
1	40/240 (16.7%)	0.81 (0.47, 1.39)	0.70 (0.42, 1.17)	0.64 (0.39, 1.07)
2+	93/324 (28.7%)	1.31 (0.80, 2.15)	1.03 (0.4, 1.65)	0.85 (0.53, 1.36)
*Trend:*		***p* = *0.036***	*p* = *0.17*	*p* = *0.84*
SLE number (negative impact only)				
Continuous		**1.20 (1.09, 1.32)** ***p* < *0.001***	**1.13 (1.03, 1.23)** ***p* = *0.007***	1.08 (0.98, 1.18) *p* = *0.11*
0	28/169 (16.6%)	1.00 [Reference]	1.00 [Reference]	1.00 [Reference]
1	49/255 (19.2%)	1.14 (0.75, 1.74)	1.07 (0.71, 1.61)	0.86 (0.59, 1.26)
2+	71/217 (32.7%)	**1.74 (1.17, 2.60)**	1.45 (1.00, 2.11)	1.16 (0.81, 1.65)
*Trend:*		***p* = *0.003***	***p* = *0.020***	*p* = *0.28*
SLE load				
Continuous		**1.18 (1.06, 1.30)** ***p* = *0.002***	1.10 (0.99, 1.21) *p* = *0.065*	1.05 (0.95, 1.16) *p* = *0.32*
0–28	32/167 (19.2%)	1.00 [Reference]	1.00 [Reference]	1.00 [Reference]
> 28–57	26/154 (16.9%)	0.82 (0.51, 1.30)	0.75 (0.48, 1.18)	0.58 (0.37, 0.89)
> 57–97	38/166 (22.9%)	1.10 (0.72, 1.67)	0.88 (0.59, 1.31)	0.72 (0.49, 1.04)
> 97–415	52/154 (33.8%)	**1.50 (1.01, 2.21)**	1.25 (0.86, 1.81)	1.03 (0.73, 1.46)
*Trend:*		***p* = *0.020***	*p* = *0.13*	*p* = *0.55*
SLE load (negative impact only)				
Continuous		**1.20 (1.08, 1.33)** ***p* = *0.001***	**1.11 (1.01, 1.23)** ***p* = *0.038***	1.07 (0.97, 1.18) *p* = *0.19*
0	28/169 (16.6%)	1.00 [Reference]	1.00 [Reference]	1.00 [Reference]
> 0–46	37/191 (19.4%)	1.16 (0.74, 1.81)	1.13 (0.73, 1.75)	0.89 (0.59, 1.33)
> 46–83	25/119 (21.0%)	1.22 (0.76, 1.98)	1.10 (0.71, 1.73)	0.91 (0.59, 1.39)
> 83–388	58/162 (35.8%)	**1.86 (1.23, 2.82)**	**1.49 (1.01, 2.19)**	1.20 (0.83, 1.73)
*Trend:*		***p* = *0.002***	***p* = *0.031***	*p* = *0.26*
You yourself suffered a serious illness				
No	148/755 (19.6%)	1.00 [Reference]	1.00 [Reference]	1.00 [Reference]
Yes	45/102 (44.1%)	**2.01 (1.53, 2.64)** ***p* < *0.001***	**1.46 (1.15, 1.87)** ***p* = *0.002***	**1.28 (1.02, 1.61)** ***p* = *0.034***
You yourself suffered a serious illness (negative only)				
No	151/760 (19.9%)	1.00 [Reference]	1.00 [Reference]	1.00 [Reference]
Yes	42/97 (43.3%)	**2.00 (1.51, 2.64)** ***p* < *0.001***	**1.44 (1.12, 1.85)** ***p* = *0.004***	**1.26 (1.00, 1.59)** ***p* = *0.049***
You became engaged, married or resumed a steady relationship (negative impact only)				
No	190/853 (22.3%)	1.00 [Reference]	1.00 [Reference]	1.00 [Reference]
Yes	3/4 (75.0%)	**4.10 (2.28, 7.38)** ***p* < *0.001***		**3.00 (1.03, 8.80)** ***p* = *0.045***
You gained a new family member (new baby born or parent remarried) (negative impact only)				
No	190/851 (22.3%)	1.00 [Reference]	1.00 [Reference]	1.00 [Reference]
Yes	3/6 (50.0%)	**2.73 (1.21, 6.16)** ***p* = *0.016***	**3.74 (2.10, 6.67)** ***p* < *0.001***	**2.46 (1.37, 4.43)** ***p* = *0.003***

All analyses by log-binomial regression

Results in boldface denote statistical significance (*p* < 0.05)

PHQ-9, Patient Health Questionnaire-9; aPR, adjusted prevalence ratio; SLE, stressful life event

^a^Model 1 adjusted for whether participants were experiencing ongoing symptoms due to a recent relapse

^b^Model 2 further adjusted for age, sex, P-MSSS, and prescription antidepressant medication

^c^Model 3 further adjusted for age, sex, P-MSSS, prescription antidepressant medication, and clinically significant fatigue

Examining individual SLEs separately found serious illness was associated with a 1.3-fold higher prevalence of depression symptoms following model 3 adjustment. Negatively impacting SLEs, including starting a serious relationship and gaining a new family member were associated with 3.0- and 2.5-fold higher depression prevalence, respectively. Individual SLEs not associated with depression following model 3 adjustment are presented in Supplementary Table 5.

### SLEs and clinically significant fatigue

SLE number and load were associated with higher fatigue prevalence (Table 3; model 1 and 2 adjustment). However, further adjustment for depressive symptoms (model 3 adjustment) deemed associations between SLEs and fatigue no longer significant, indicating SLEs acting via depressive symptoms were likely to be responsible for the observed SLE-fatigue relationship.

**Table 3:** SLE characteristics of clinically significant fatigue

SLE measure	n/N (row %)	aPR (95% CI)^a^	aPR (95% CI)^b^	aPR (95% CI)^c^
SLE number				
Continuous		**1.07 (1.00, 1.14)** ***p* = *0.038***	1.06 (1.00, 1.13) *p* = *0.071*	1.03 (0.96, 1.09) *p* = *0.43*
0	22/78 (28.2%)	1.00 [Reference]	1.00 [Reference]	1.00 [Reference]
1	94/242 (38.8%)	1.34 (0.91, 1.98)	1.27 (0.87, 1.85)	1.33 (0.93, 1.93)
2+	144/321 (44.9%)	**1.52 (1.04, 2.21)**	1.34 (0.92, 1.94)	1.27 (0.89, 1.82)
*Trend:*		***p* = *0.019***	***p* = *0.041***	*p* = *0.17*
SLE number (negative impact only)				
Continuous		**1.11 (1.03, 1.19)** ***p* = *0.005***	**1.09 (1.01, 1.17)** ***p* = *0.023***	1.04 (0.97, 1.12) *p* = *0.26*
0	51/172 (29.7%)	1.00 [Reference]	1.00 [Reference]	1.00 [Reference]
1	110/255 (43.1%)	**1.43 (1.09, 1.87)**	**1.31 (1.01, 1.71)**	**1.36 (1.05, 1.77)**
2+	99/214 (46.3%)	**1.47 (1.11, 1.94)**	1.27 (0.97, 1.68)	1.18 (0.90, 1.54)
*Trend:*		***p* = *0.007***	***p* = *0.036***	*p* = *0.18*
SLE load				
Continuous		**1.11 (1.03, 1.20)** ***p* = *0.005***	**1.08 (1.00, 1.16)** ***p* = *0.041***	1.04 (0.97, 1.12) *p* = *0.26*
0–28	51/170 (30.0%)	1.00 [Reference]	1.00 [Reference]	1.00 [Reference]
> 28–57	67/156 (43.05)	**1.37 (1.02, 1.83)**	1.23 (0.92, 1.64)	**1.34 (1.02, 1.78)**
> 57–97	74/166 (44.6%)	**1.44 (1.08, 1.92)**	1.23 (0.92, 1.63)	1.26 (0.96, 1.66)
> 97–415	68/149 (45.6%)	**1.39 (1.04, 1.87)**	1.20 (0.90, 1.61)	1.14 (0.87, 1.50)
*Trend:*		***p* = *0.021***	*p* = *0.12*	*p* = *0.26*
SLE load (negative impact only)				
Continuous		**1.13 (1.04, 1.22)** ***p* = *0.003***	**1.09 (1.01, 1.18)** ***p* = *0.027***	1.05 (0.98, 1.13) *p* = *0.20*
0	51/172 (29.7%)	1.00 [Reference]	1.00 [Reference]	1.00 [Reference]
> 0–46	83/193 (43.0%)	**1.44 (1.08, 1.91)**	**1.33 (1.01, 1.75)**	**1.35 (1.03, 1.77)**
> 46–83	49/120 (40.8%)	1.33 (0.97, 1.83)	1.26 (0.93, 1.72)	1.26 (0.94, 1.72)
> 83–388	77/156 (49.4%)	**1.55 (1.16, 2.06)**	1.28 (0.96, 1.71)	1.20 (0.91, 1.58)
*Trend:*		***p* = *0.007***	*p* = *0.068*	*p* = *0.22*
You yourself suffered a serious illness				
No	293/759 (38.6%)	1.00 [Reference]	1.00 [Reference]	1.00 [Reference]
Yes	59/99 (59.6%)	**1.45 (1.19, 1.76)** ***p* < *0.001***	**1.26 (1.05, 1.52)** ***p* = *0.015***	1.10 (0.91, 1.32) *p* = *0.31*
You yourself suffered a serious illness (negative only)				
No	297/765 (38.8%)	1.00 [Reference]	1.00 [Reference]	1.00 [Reference]
Yes	55/93 (59.1%)	**1.45 (1.19, 1.77)** ***p* < *0.001***	**1.29 (1.07, 1.56)** ***p* = *0.007***	1.12 (0.93, 1.35) *p* = *0.22*
You became engaged married or resumed a steady relationship (negative impact only)				
No	350/855 (40.9%)	1.00 [Reference]	1.00 [Reference]	1.00 [Reference]
Yes	2/3 (66.7%)	1.77 (0.79, 3.97) *p* = *0.17*	**2.31 (1.05, 5.06)** ***p* = *0.037***	1.60 (0.57, 4.44) *p* = *0.37*

All analyses by log-binomial regression

Results in boldface denote statistical significance (*p* < 0.05)

aPR, adjusted prevalence ratio; SLE, stressful life event

^a^Model 1 adjusted for whether participants were experiencing ongoing symptoms due to a recent relapse

^b^Model 2 further adjusted for Model 1 adjustments and age, sex, P-MSSS, prescription antifatigue medication, and baseline comorbidity number

^c^Model 3 adjusted for Model 2 adjustment and depressive symptoms (PHQ-9)

Serious illness and a new relationship with a negative impact were associated with 26% and 2.3-fold higher fatigue prevalence on model 2 adjustment, but this association was no longer significant after adjusting for depressive symptoms (model 3 adjustment). Other SLEs not associated with fatigue following model 2 adjustment are presented in Supplementary Table 6.

## Discussion

We identified associations between certain SLEs and health outcomes in PlwMS. Major findings included highlighting the association between certain SLEs with a perceived negative emotional impact were plausible risk factors for depressive symptoms; a new relationship (engagement/marriage/steady relationship) and new family member (birth of baby/parent remarried) were associated with a threefold and 2.5-fold higher incidence of depressive symptoms, as well as serious illness and 28% higher prevalence of depressive symptoms. Notably, SLE number or load were not associated with any health outcomes examined. Overall, study findings highlight the saliency of specific SLEs in understanding disease and mental-health prognostic factors in PlwMS.

### SLEs and depressive symptoms

Clinical levels of depression are highly prevalent among plwMS (30–50%) [24], and have been linked to poor QoL [25], and negative prognostic factors (eg fatigue) [26]. However, depression is frequently under-recognized and undertreated in plwMS in clinical care, resulting in inadequate therapy for depression [14]. Clinical management of depression is further complicated by symptom overlap with fatigue, making the two conditions difficult to distinguish [27]. Despite evidence of biological aetiologies for depression in plwMS [28], our results support the contribution of psychological causes of depression [29], in particular family/relationship situations perceived to have a negative impact. These associations persisted despite adjusting for fatigue in the multivariable model, indicating SLEs may be directly associated with depressive symptoms.

Assessment for symptoms of depression in those experiencing serious illness may be important due to the identified associations of a 26–28% higher prevalence of depressive symptoms in those with a serious illness. However, as the nature of the serious illness was not queried [17], this finding prompts the need for future prospective studies to investigate potential associations between different forms of serious illness and depression.

Potential mechanisms for the associations between serious illness and depression in plwMS include the pivotal role that inflammation has been found to play in depression [30]. That is, systemic bacterial and viral respiratory infections facilitating increased inflammatory responses [31, 32], may be responsible for promoting depression in plwMS by facilitating increased inflammatory activity. However, as infections are often acute, treatable conditions, they are unlikely to be responsible for long-term depression in plwMS.

Alternatively, ‘serious illness’ could indicate the presence of a chronic comorbidity such as cardiovascular disease or cancer. Therefore, the chronic condition itself (that is, disease symptoms, treatment side effects or poor prognosis) may directly result in depressive symptoms in plwMS and in certain cases, the impact of a chronic disease on depression may be long-term.

Overall, probing for certain SLEs in clinical practice particularly changes in personal/relationships with a negative impact or serious illness, may be beneficial for identifying and treating depressive symptoms. Further, early identification of SLEs could facilitate timely cognitive behavioral intervention (i.e., re-structuring, problem-solving) and mindfulness strategies to lessen the impacts of stress and the overall burden of depression. Through cognitive restructuring, overly distressing or catastrophic interpretations of SLEs contributing to depressive symptoms may be altered, possibly reducing ongoing effects of SLEs.

### SLEs and fatigue

Fatigue is a common symptom of MS, regarded as one of the most troubling and significantly interfering with QoL [33]. By adopting recommendations for determining clinically significant fatigue levels (FSS > 5) [21], 41% of participants were characterized with clinical levels of fatigue compared with previous estimates (75–90%) [33]. These differences may be attributable to differences in sample characteristics (age/baseline MS type), and/or subjective fatigue measurement tools. Alternately, indications suggest our analysis sample may be healthier than the general MS population with participants with severe fatigue not retained in the study.

As the nature of fatigue is poorly understood and difficult to treat, possibly due to reciprocal relationships with disease factors (e.g., proinflammatory cytokines, hypothalamic–pituitary–adrenal axis dysregulation) and challenges disentangling fatigue from cognitive impairment and depression [34], identifying risk factors for fatigue in PlwMS is critical for improving clinical understanding and management. We found SLEs alone (their nature, the number of events and their perceived impact) did not represent plausible risk factors for fatigue, with analyses including adjusting for depression obliterating SLE-fatigue associations suggesting SLEs may be linked to symptoms of depression, not fatigue. While SLEs could be acting via primary causes of depression (and fatigue) including frequently-reported anhedonia or secondary causes including pain, certain disease-modifying therapies (DMTs) and sleep disorders [35], this was beyond the scope of our study but may be beneficial in future prospective studies.

### Stress-reduction techniques

Stress is associated with reduced wellbeing, self-management, and QoL in plwMS [12], and possibly depression (our findings), highlighting the potential value of stress-reduction interventions for improving MS symptomology and QoL. Studies indicate physiological and psychological stress in plwMS may be effectively modified through stress management therapies. In randomised controlled trials (RCTs), psychologist-led stress management sessions over 24 weeks reduced new brain lesions in individuals with RRMS [36], and cognitive behaviour therapy and relaxation therapy improved fatigue in plwMS [37], with mindfulness improving QoL, depression, and fatigue [38]. With the proven efficacy of stress-reducing activities and minimal associated side effects, recommendations to include stress management therapies in clinical care appear well-justified.

### Strengths and limitations

Strengths include the large international cohort with demographic and clinical characteristics comparable to other MS cohorts [39]. However, the immunomodulatory treatment rate in participants was low (37%) which may reflect the recruitment bias in our sample. This may indicate a representativeness issue in this aspect of our study cohort but in all other respects, our cohort represents as clinically and demographically typical of plwMS. Validated self-reported tools for measuring depressive symptoms, and fatigue in plwMS were utilised; PHQ-9 has been found to be a valid measure of depressive symptoms in plwMS with both external and internal reliability [20, 40], with FSS also demonstrated to be a reliable and valid tool for measuring clinically significant fatigue in plwMS [21, 41, 42].

Overall, our study findings provide context to the differential impact of SLE type and severity on health outcomes in MS, extending previous research indicating potentially adverse effects of SLEs [43, 44]. Our study also provides support to perform future studies to confirm the findings of the present study and examine associations between SLEs and other health outcomes such as disability and relapse, which preferentially have been clinically evaluated.

Future studies may also wish to examine clinically evaluated measures such as MS type, as this was self-reported in HOLISM (50). Moreover, MS type was also only queried at baseline and does not reflect the MS type of participants at the 7.5-year follow-up. MS type at 7.5 years would have been a more appropriate confounder to adjust for in analyses examining characteristics associated with SLE number, however, MS type was not queried after baseline. Further, future studies should endeavour to adjust for specific DMT usage as certain DMTs have the potential to improve inflammation, and other health outcomes, including depression [27]. However, data on individual DMTs was not available for the present study.

Given the interplay between SLEs and health outcomes in plwMS is complex, it is important to recognize that associations identified in the present study may also be partially due to the effect of extraneous variables. For instance, data was not available to examine factors linked to depression in plwMS, including unemployment, low self-efficacy, poor social support and cognitive impairment [45]. Moreover, participants with other psychiatric disorders (trauma-related/anxiety/substance-use) were also not screened for or excluded which may affect the likelihood of SLEs. For instance, in generalised anxiety disorder there may be hypervigilance to SLEs and increased likelihood to appraise SLEs as overly threatening.

It is likely selection bias occurred due to attrition and significantly fewer participants presenting with depressive symptoms, fatigue and ≥ 2 comorbidities at 7.5 years compared with baseline, indicating people with depression, fatigue, or unwell/impaired by MS symptoms may have been more likely to drop out. Subsequently, the analysis sample most likely comprised a healthier cohort with less depression and fatigue symptoms, or a more help-seeking cohort with access to social and medical resources than the general MS community which may explain why associations with certain health outcomes were not identified.

As cross-sectional associations between SLEs and health outcomes were examined, only a snapshot was examined and the temporality of events was not established, precluding a causal relationship. Moreover, the possibility of reverse causality exists, with MS symptoms examined associated with increased stress [46]. While we chose to examine SLEs individually to identify potential drivers of associations with health outcomes, a statistical method to counteract problems associated with multiple comparisons such as Bonferroni correction [47], was not used due to its stricter significance threshold for individual comparisons to compensate for multiple interferences due to increased likelihood of type I error.

Although SLE recall error/bias is unlikely for significant SLEs including divorce/death, potential recall bias may exist for less significant SLEs. Further, examining cognitive function was beyond the scope of the study but serious impairment may have confounded recall of SLEs.

## Conclusions

Our study findings offer a platform for discourse on the potential of SLEs to adversely affect health outcomes in PlwMS, and SLE appraisal as a tenable target for psychological intervention. We highlight the association between serious illness and relationship-related SLEs with a perceived negative impact and increased depression prevalence. Future longitudinal research is recommended to replicate study findings and clarify temporal effects between SLEs and health outcomes.

### Supplementary Information

Below is the link to the electronic supplementary material.

Supplementary file1 (DOCX 59 KB)

## Data Availability

Data is unable to be freely shared due to the conditions approved by the University of Melbourne ethics committee, in that all data are stored as reidentifiable information in the form of password-protected computer databases, and only the listed investigators have access to the data. All data have been reported on a group basis, summarising the group findings rather than individual findings so personal information cannot be identified.
