# Supplementary material for: Stressful life events and depression and fatigue in people with multiple sclerosis: a cross-sectional analysis of an international cohort
Source: Acta Neurol Belg. 2023 Sep 28;124(2):457–66. doi: 10.1007/s13760-023-02390-z (PMC10965589; doi:10.1007/s13760-023-02390-z)
Supplement: Supplementary file 1 — Supplementary file1 (DOCX 59 KB) [file 13760_2023_2390_MOESM1_ESM.docx]

Supplementary Table 1. SLEs and weights.

| SLE | Weight |
| --- | --- |
| Your parent, child or partner died | 100 |
| You (or your parents) broke off a steady relationship | 69 |
| You yourself suffered a serious illness | 57 |
| You yourself suffered a serious injury or assault | 57 |
| A serious illness, injury or assault happened to a close relative or friend | 46 |
| You had a major financial crisis | 43 |
| A close family friend or another relative died | 40 |
| You became engaged married or resumed a steady relationship | 39 |
| You were fired/sacked from your job or expelled from school | 34 |
| You had an outstanding personal achievement (awards, grades, etc.) | 28 |
| You became pregnant or menopausal | 27 |
| You changed your personal habits, including use of alcohol or drugs | 26 |
| You had a crisis or serious disappointment in your work, school or career | 22 |
| You changed house, school, or jobs | 22 |
| You gained a new family member (new baby born or parent remarried) | 21 |
| You had problems with the police and a court appearance | 15 |
| SLE = Stressful Life Events. Weights from Scully and colleagues^15^. |  |

Supplementary Table 2. Power calculations for study analyses

|  | Proportion with outcome | Proportion exposed | Proportion with outcome among exposed | Proportion with outcome among nonexposed | Power |
| --- | --- | --- | --- | --- | --- |
| *2+ SLE vs none* |  |  |  |  |  |
| Fatigue | 41.0% | 50.2% | 44.9% | 28.2% | 100.0% |
| Depression | 22.5% | 50.2% | 28.7% | 19.5% | 93.4% |
| *Participant experiences illness* |  |  |  |  |  |
| Fatigue | 41.0% | 11.1% | 59.6% | 38.6% | 98.6% |
| Depression | 22.5% | 11.1% | 44.1% | 19.6% | 99.9% |

Supplementary Table 3. Baseline cohort characteristics of participants retained at 7.5-year review

compared with those lost to follow-up.

| **Characteristic** | **Lost to follow-up**  **(n=1518)** | **Analysis sample**  **(n=948)** |  |
| --- | --- | --- | --- |
| Sex  Male  Female | 230 (16.1%)  1196 (83.9%)  (92 (6.1%)) | 187 (19.8%)  760 (80.2%)  (0 (0%)) | **p=0.024**  - |
| MS type |  |  |  |
| RRMS  SPMS  PPMS  PRMS  Unsure  (Missing) | 912 (61.5%)  195 (13.2%)  118 (8.0%)  38 (2.6%)  219 (14.8%)  (36 (2.4%)) | 679 (72.5%)  80 (8.5%)  57 (6.1%)  10 (1.1%)  111 (11.9%)  (11 (1.2%)) | **p<0.001**  **p=0.011**  **p=0.004**  **p=0.002**  **p=0.011** |
| Clinically significant fatigue |  |  |  |
| No  Yes  (Missing) | 614 (48.3%)  657 (51.7%)  (247 (16.3%)) | 517 (59.6%)  350 (40.4%)  (81 (8.5%)) | **p<0.001**  **p<0.001** |
| Depression (PHQ-2) |  |  |  |
| No  Yes  (Missing) | 1007 (76.5%  310 (23.5%)  (201 (13.2%)) | 792 (87.3%)  115 (12.7%)  (41 (4.3%)) | **p<0.001**  **p<0.001** |
| Baseline treated comorbidity number |  |  |  |
| 0  1  ≥2 | 802 (52.8%)  355 (23.4%)  361 (23.8%) | 573 (60.4%)  219 (23.1%)  156 (16.5%) | p=0.15  **p<0.001** |
| Immunomodulatory medication |  |  |  |
| No  Yes | 851 (56.1%)  667 (43.9%) | 470 (49.6%)  478 (50.4%) | **p=0.002** |
| Prescription antidepressant medication |  |  |  |
| No  Yes | 1173 (77.3%)  345 (22.7%) | 791 (83.4%)  157 (16.6%) | **p<0.001** |
| Prescription antifatigue medication |  |  |  |
| No  Yes | 1359 (89.5%)  159 (10.5%) | 870 (91.9%)  78 (8.2%) | p=0.066 |
|  |  |  |  |
| Age | 46.0 (10.6; 18.0-78.5) | 45.2 (10.2; 18.0-79.0) | p=0.052 |
|  |  |  |  |
| P-MSSS | 2.7 (0.7-5.3) | 1.3 (0.5-4.4) | **p<0.001** |
| Duration since MS onset, years | 12.4 (6.4-21.5) | 10.6 (5.5-18.5) | **p=0.002** |
| Abbreviations: IQR = interquartile range; PHQ-2 = Patient Health Questionnaire-2; P-MSSS = Patient-reported MS Severity Score; PPMS = primary progressive MS; PRMS = progressive relapsing MS; RRMS = relapsing-remitting MS; SPMS = secondary progressive MS. | | |  |

Supplementary Table 4. Characteristics of SLE number at 7.5-year review.

| **SLE measure** |  |  | **PR (95% CI)** | **aPR (95% CI)** | **PR (95% CI)** | **aPR (95% CI)** |
| --- | --- | --- | --- | --- | --- | --- |
|  | **1** | **≥2** | **1 vs 0** |  | **≥2 vs 0** |  |
| Sex  Male  Female | 52/129 (40.3%)  197/532 (37.0%) | 55/129 (42.6%)  277/532 (52.1%) | 1.00 [Reference]  1.10 (0.93, 1.29)  *p=0.25* | 1.00 [Reference]  1.07 (0.90, 1.27)  *p=0.43* | 1.00 [Reference]  1.16 (1.00, 1.34)  *p=0.055* | 1.00 [Reference]  1.14 (0.98, 1.34)  *p=0.093* |
| Age, years |  |  |  |  |  |  |
| 25.4 – 44.8  >44.8 – 52.6  >52.6 – 60.0  >60.0 – 86.6  *Trend:* | 51/163 (31.3%)  47/163 (28.8%)  73/174 (42.0%)  78/161 (48.5%) | 91/163 (55.8%)  101/163 (62.0%)  78/174 (44.8%)  62/161 (38.5%) | 1.00 [Reference]  1.07 (0.87, 1.31)  1.07 (0.89, 1.29)  1.11 (0.93, 1.33)  *p=0.26* | 1.00 [Reference]  1.05 (0.85, 1.29)  1.04 (0.86, 1.26)  1.12 (0.92, 1.35)  *p=0.17* | 1.00 [Reference]  1.07 (0.96, 1.20)  0.95 (0.83, 1.09)  0.92 (0.79, 1.07)  *p=0.13* | 1.00 [Reference]  1.02 (0.91, 1.15)  0.95 (0.82, 1.09)  0.92 (0.79, 1.07)  *p=0.19* |
| Education |  |  |  |  |  |  |
| Secondary or less  Vocational  Bachelor’s  Postgraduate  *Trend:* | 27/65 (41.5%)  47/113 (41.6%)  87/247 (35.2%)  87/235 (37.0%) | 35/65 (53.9%)  47/113 (41.6%)  125/247 (50.6%)  125/235 (53.2%) | 1.00 [Reference]  **0.79 (0.65, 0.96)**  **0.79 (0.67, 0.93)**  0.88 (0.75, 1.02)  *p=0.78* | 1.00 [Reference]  0.82 (0.67, 1.00)  0.81 (0.68, 0.96)  0.91 (0.26, 1.08)  *p=0.92* | 1.00 [Reference]  **0.77 (0.65, 0.93)**  **0.85 (0.75, 0.96)**  0.92 (0.82, 1.03)  *p=0.65* | 1.00 [Reference]  **0.80 (0.67, 0.95)**  **0.87 (0.77, 0.99)**  0.93 (0.83, 1.04)  *p=0.66* |
| Employment status |  |  |  |  |  |  |
| Employed  Not employed  Retired, age  Retired, disability | 134/373 (35.9%)  26/79 (32.9%)  24/51 (47.1%)  64/156 (41.0%) | 193/373 (51.7%)  43/79 (54.4%)  19/51 (37.3%)  76/156 (48.7%) | 1.00 [Reference]  0.97 (0.78, 1.21)  1.01 (0.81, 1.25)  1.07 (0.93, 1.24) | 1.00 [Reference]  0.97 (0.77, 1.23)  0.91 (0.71, 1.17)  0.99 (0.84, 1.18) | 1.00 [Reference]  1.00 (0.87, 1.16)  0.87 (0.68, 1.12)  1.02 (0.91, 1.14) | 1.00 [Reference]  0.95 (0.81, 1.10)  1.04 (0.81, 1.35)  1.03 (0.89, 1.19) |
| MS type (baseline) |  |  |  |  |  |  |
| RRMS  SPMS  PPMS  PRMS  Unsure | 183/489 (37.4%)  21/52 (40.4%)  15/27 (55.6%)  3/6 (50.0%)  26/80 (32.5%) | 251/489 (51.3%)  18/52 (34.6%)  9/27 (33.3%)  3/6 (50.0%)  46/80 (57.5%) | 1.00 [Reference]  0.80 (0.61, 1.06)  1.08 (0.87, 1.35)  **1.30 (1.21, 1.39)**  0.99 (0.81, 1.21) | 1.00 [Reference]  0.77 (0.57, 1.02)  0.98 (0.73, 1.32)  1.08 (0.87, 1.33)  1.04 (0.84, 1.27) | 1.00 [Reference]  **0.71 (0.52, 0.96)**  0.91 (0.66, 1.27)  **1.22 (1.16, 1.28)**  1.04 (0.92, 1.17) | 1.00 [Reference]  **0.71 (0.53, 0.96)**  0.88 (0.63, 1.23)  1.02 (0.85, 1.21)  1.09 (0.97, 1.23) |
| Duration since MS onset, years |  |  |  |  |  |  |
| 8.0 – 13.0  >13.0 – 18.1  >18.1 – 26.1  >26.1 – 56.1  *Trend:* | 63/175 (36.0%)  54/166 (32.5%)  63/148 (42.6%)  69/172 (40.1%) | 90/175 (51.4%)  90/166 (54.2%)  67/148 (45.3%)  85/172 (49.4%) | 1.00 [Reference]  0.96 (0.79, 1.16)  1.05 (0.88, 1.25)  1.07 (0.91, 1.26)  *p=0.29* | 1.00 [Reference]  0.94 (0.77, 1.14)  1.02 (0.86, 1.22)  1.09 (0.91, 1.30)  *p=0.28* | 1.00 [Reference]  1.00 (0.88, 1.14)  0.98 (0.85, 1.13)  1.03 (0.90, 1.17)  *p=0.76* | 1.00 [Reference]  0.98 (0.86, 1.12)  0.98 (0.84, 1.15)  1.09 (0.94, 1.27)  *p=0.18* |
| P-MSSS |  |  |  |  |  |  |
| Normal/mild  Moderate  Severe  *Trend:* | 170/451 (37.7%)  50/140 (35.7%)  28/68 (41.2%) | 222/451 (49.2%)  80/140 (57.1%)  29/68 (42.7%) | 1.00 [Reference]  1.12 (0.98, 1.29)  0.97 (0.78, 1.19)  *p=0.76* | 1.00 [Reference]  1.11 (0.98, 1.27)  0.99 (0.78, 1.26)  *p=0.66* | 1.00 [Reference]  **1.13 (1.02, 1.24)**  0.92 (0.75, 1.12)  *p=0.85* | 1.00 [Reference]  **1.16 (1.06, 1.26)**  0.93 (0.75, 1.17)  *p=0.52* |
| Clinically significant fatigue |  |  |  |  |  |  |
| No  Yes | 148/381 (38.9%)  94/260 (36.2%) | 177/381 (46.5%)  144/260 (55.4%) | 1.00 [Reference]  1.12 (0.99, 1.26)  *p=0.076* | 1.00 [Reference]  1.11 (0.98, 1.27)  *p=0.10* | 1.00 [Reference]  **1.14 (1.04, 1.25)**  ***p=0.005*** | 1.00 [Reference]  **1.13 (1.03, 1.25)**  ***p=0.014*** |
| Depression (PHQ-9) |  |  |  |  |  |  |
| No  Yes | 200/493 (40.6%)  40/148 (27.0%) | 231/493 (46.9%)  93/148 (62.8%) | 1.00 [Reference]  0.95 (0.80, 1.14)  *p=0.59* | 1.00 [Reference]  0.87 (0.72, 1.05)  *p=0.15* | 1.00 [Reference]  1.09 (0.99, 1.20)  *p=0.073* | 1.00 [Reference]  1.02 (0.90, 1.15)  *p=0.78* |
| Diagnosed comorbidity number |  |  |  |  |  |  |
| 0  1  2+  *Trend:* | 191/500 (38.2%)  27/72 (37.5%)  31/89 (34.8%) | 242/500 (48.4%)  36/72 (50.0%)  54/89 (60.7%) | 1.00 [Reference]  1.01 (0.83, 1.24)  **1.20 (1.04, 1.38)**  ***p=0.034*** | 1.00 [Reference]  0.98 (0.80, 1.19)  1.15 (0.97, 1.35)  *p=0.25* | 1.00 [Reference]  1.02 (0.87, 1.20)  **1.19 (1.08, 1.30)**  ***p=0.001*** | 1.00 [Reference]  1.00 (0.86, 1.16)  1.09 (0.97, 1.22)  *p=0.080* |
| Prescription antidepressant medication |  |  |  |  |  |  |
| No  Yes | 195/500 (39.0%)  54/161 (33.5%) | 237/500 (47.4%)  95/161 (59.0%) | 1.00 [Reference]  1.10 (0.96, 1.26)  *p=0.15* | 1.00 [Reference]  1.13 (0.98, 1.30)  *p=0.088* | 1.00 [Reference]  **1.14 (1.04, 1.25)**  ***p=0.004*** | 1.00 [Reference]  **1.12 (1.02, 1.23)**  ***p=0.019*** |
| Prescription antifatigue medication |  |  |  |  |  |  |
| No  Yes | 228/598 (38.1%)  21/63 (33.3%) | 296/598 (49.5%)  36/63 (57.1%) | 1.00 [Reference]  1.03 (0.83, 1.27)  *p=0.78* | 1.00 [Reference]  1.01 (0.82, 1.25)  *p=0.89* | 1.00 [Reference]  1.07 (0.94, 1.22)  *p=0.31* | 1.00 [Reference]  1.03 (0.91, 1.18)  *p=0.63* |
| All analyses by log-multinomial regression.  Adjusted models include age, sex, education, baseline MS type, P-MSSS, clinically significant fatigue, and prescription antidepressant medication.  Results in boldface denote statistical significance (p<0.05).  Abbreviations: IQR = interquartile range; PHQ-9 = Patient Health Questionnaire-9; P-MSSS = Patient-reported MS Severity Score; PPMS = primary progressive MS; aPR = adjusted prevalence ratio; PRMS = progressive relapsing MS; RRMS = relapsing-remitting MS; SLE = stressful life event; SPMS = secondary progressive MS. | | | | | | |

Supplementary Table 5. Individual SLEs **not** associated with depression (PHQ-9) at 7.5-year review.

| **SLE measure** | **n/N (row %)** | **aPR (95% CI)^a^** | **aPR (95% CI)^b^** | **aPR (95% CI)^c^** |
| --- | --- | --- | --- | --- |
| Your parent, child or partner died |  |  |  |  |
| No  Yes | 180/804 (22.4%)  13/53 (24.5%) | 1.00 [Reference]  1.03 (0.63, 1.69)  *p=0.90* | 1.00 [Reference]  0.95 (0.58, 1.53)  *p=0.82* | 1.00 [Reference]  1.00 (0.67, 1.47)  *p=0.98* |
| Your parent, child or partner died (negative impact only) |  |  |  |  |
| No  Yes | 181/808 (22.4%)  12/49 (24.5%) | 1.00 [Reference]  1.02 (0.61, 1.71)  *p=0.94* | 1.00 [Reference]  0.96 (0.58, 1.60)  *p=0.88* | 1.00 [Reference]  1.00 (0.66, 1.52)  *p=0.99* |
| A close family friend or another relative died |  |  |  |  |
| No  Yes | 156/689 (22.6%)  37/168 (22.0%) | 1.00 [Reference]  0.94 (0.68, 1.30)  *p=0.71* | 1.00 [Reference]  0.86 (0.64, 1.15)  *p=0.31* | 1.00 [Reference]  0.82 (0.62, 1.08)  *p=0.15* |
| A close family friend or another relative died (negative impact only) |  |  |  |  |
| No  Yes | 158/706 (22.4%)  35/151 (23.2%) | 1.00 [Reference]  0.99 (0.72, 1.37)  *p=0.95* | 1.00 [Reference]  0.91 (0.67, 1.22)  *p=0.53* | 1.00 [Reference]  0.86 (0.66, 1.14)  *p=0.30* |
| You yourself suffered a serious injury or assault |  |  |  |  |
| No  Yes | 182/825 (22.1%)  11/32 (34.4%) | 1.00 [Reference]  1.47 (0.87, 2.50  *p=0.15* | 1.00 [Reference]  1.39 (0.88, 2.20)  *p=0.16* | 1.00 [Reference]  1.29 (0.84, 1.99)  *p=0.25* |
| You yourself suffered a serious injury or assault (negative impact only) |  |  |  |  |
| No  Yes | 182/827 (220%)  11/30 (36.7%) | 1.00 [Reference]  1.56 (0.92, 2.64)  *p=0.097* | 1.00 [Reference]  1.48 (0.94, 2.32)  *p=0.087* | 1.00 [Reference]  1.32 (0.85, 2.03)  *p=0.22* |
| A serious illness, injury or assault happened to a close relative or friend |  |  |  |  |
| No  Yes | 157/718 (21.9%)  36/139 (25.9%) | 1.00 [Reference]  1.12 (0.82, 1.52)  *p=0.48* | 1.00 [Reference]  1.05 (0.80, 1.39)  *p=0.71* | 1.00 [Reference]  0.93 (0.71, 1.23)  *p=0.62* |
| A serious illness, injury or assault happened to a close relative or friend (negative impact only) |  |  |  |  |
| No  Yes | 158/729 (21.7%)  35/128 (27.3%) | 1.00 [Reference]  1.18 (0.87, 1.60)  *p=0.29* | 1.00 [Reference]  1.09 (0.82, 1.44)  *p=0.55* | 1.00 [Reference]  0.94 (0.71, 1.25)  *p=0.69* |
| You (or your parents) broke off a steady relationship |  |  |  |  |
| No  Yes | 186/822 (22.6%)  7/35 (20.0%) | 1.00 [Reference]  0.91 (0.49, 1.70)  *p=0.76* | 1.00 [Reference]  0.88 (0.46, 1.70)  *p=0.71* | 1.00 [Reference]  1.07 (0.63, 1.80)  *p=0.81* |
| You (or your parents) broke off a steady relationship (negative impact only) |  |  |  |  |
| No  Yes | 187/825 (22.7%)  6/32 (18.8%) | 1.00 [Reference]  0.84 (0.43, 1.64)  *p=0.60* | 1.00 [Reference]  0.84 (0.41, 1.72)  *p=0.63* | 1.00 [Reference]  1.07 (0.60, 1.93)  *p=0.81* |
| You became pregnant or menopausal |  |  |  |  |
| No  Yes | 174/786 (22.1%)  19/71 (26.8%) | 1.00 [Reference]  1.16 (0.76, 1.77)  *p=0.49* | 1.00 [Reference]  1.18 (0.78, 1.79)  *p=0.43* | 1.00 [Reference]  1.01 (0.68, 1.49)  *p=0.96* |
| You became pregnant or menopausal (negative impact only) |  |  |  |  |
| No  Yes | 178/808 (22.0%)  15/49 (30.6%) | 1.00 [Reference]  1.29 (0.80, 2.08)  *p=0.30* | 1.00 [Reference]  1.28 (0.82, 1.98)  *p=0.28* | 1.00 [Reference]  1.07 (0.70, 1.61)  *p=0.76* |
| You became engaged married or resumed a steady relationship |  |  |  |  |
| No  Yes | 186/836 (22.3%)  7/21 (33.3%) | 1.00 [Reference]  1.56 (0.85, 2.86)  *p=0.15* | 1.00 [Reference]  1.31 (0.71, 2.44)  *p=0.39* | 1.00 [Reference]  1.23 (0.62, 2.46)  *p=0.55* |
| You had problems with the police and a court appearance |  |  |  |  |
| No  Yes | 192/850 (22.6%)  1/7 (14.3%) | 1.00 [Reference]  0.66 (0.12, 3.68)  *p=0.63* | 1.00 [Reference]  0.68 (0.09, 4.99)  *p=0.70* | 1.00 [Reference]  0.55 (0.08, 3.96)  *p=0.55* |
| You were fired/sacked from your job or expelled from school |  |  |  |  |
| No  Yes | 191/847 (22.6%)  2/10 (20.0%) | 1.00 [Reference]  0.74 (0.22, 2.51)  *p=0.63* | 1.00 [Reference]  0.99 (0.25, 3.92)  *p=0.99* | 1.00 [Reference]  1.70 (0.60, 4.80)  *p=0.32* |
| You were fired/sacked from your job or expelled from school (negative impact only) |  |  |  |  |
| No  Yes | 191/848 (22.5%)  2/9 (22.2%) | 1.00 [Reference]  0.80 (0.24, 2.69)  *p=0.72* | 1.00 [Reference]  1.06 (0.27, 4.21)  *p=0.94* | 1.00 [Reference]  1.79 (0.63, 5.05)  *p=0.27* |
| You had a crisis or serious disappointment in your work, school or career |  |  |  |  |
| No  Yes | 163/749 (21.8%)  30/108 (27.8%) | 1.00 [Reference]  1.21 (0.87, 1.69)  *p=0.25* | 1.00 [Reference]  1.22 (0.89, 1.66)  *p=0.22* | 1.00 [Reference]  1.28 (0.94, 1.74)  *p=0.12* |
| You had a crisis or serious disappointment in your work, school or career (negative impact only) |  |  |  |  |
| No  Yes | 165/755 (21.9%)  28/102 (27.5%) | 1.00 [Reference]  1.21 (0.86, 1.70)  *p=0.28* | 1.00 [Reference]  1.23 (0.89, 1.70)  *p=0.21* | 1.00 [Reference]  1.31 (0.96, 1.79)  *p=0.092* |
| You gained a new family member (new baby born or parent remarried) |  |  |  |  |
| No  Yes | 177/791 (22.4%)  16/66 (24.2%) | 1.00 [Reference]  1.12 (0.72, 1.75)  *p=0.61* | 1.00 [Reference]  0.93 (0.61, 1.43)  *p=0.75* | 1.00 [Reference]  0.85 (0.57, 1.27)  *p=0.44* |
| You changed house, school, or jobs |  |  |  |  |
| No  Yes | 162/731 (22.2%)  31/126 (24.6%) | 1.00 [Reference]  1.09 (0.78, 1.53)  *p=0.61* | 1.00 [Reference]  1.15 (0.84, 1.56)  *p=0.39* | 1.00 [Reference]  1.25 (0.94, 1.67)  *p=0.12* |
| You changed house, school, or jobs (negative impact only) |  |  |  |  |
| No  Yes | 175/801 (21.9%)  18/56 (32.1%) | 1.00 [Reference]  1.32 (0.87, 1.99)  *p=0.19* | 1.00 [Reference]  **1.49 (1.01, 2.20)**  ***p=0.044*** | 1.00 [Reference]  1.43 (0.98, 2.09)  *p=0.067* |
| You changed your  personal habits, including use of alcohol or drugs (negative impact only) |  |  |  |  |
| No  Yes | 187/842 (22.2%)  6/15 (40.0%) | 1.00 [Reference]  1.71 (0.97, 3.02)  *p=0.061* | 1.00 [Reference]  1.42 (0.96, 2.09)  *p=0.081* | 1.00 [Reference]  1.25 (0.61, 2.56)  *p=0.55* |
| You had a major financial crisis |  |  |  |  |
| No  Yes | 170/797 (21.3%)  23/60 (38.3%) | 1.00 [Reference]  **1.71 (1.23, 2.39)**  ***p=0.002*** | 1.00 [Reference]  1.30 (0.95, 1.77)  *p=0.098* | 1.00 [Reference]  1.29 (0.98, 1.70)  *p=0.067* |
| You had a major financial crisis (negative impact only) |  |  |  |  |
| No  Yes | 171/800 (21.4%)  22/57 (38.6%) | 1.00 [Reference]  **1.70 (1.21, 2.39)**  ***p=0.002*** | 1.00 [Reference]  1.31 (0.95, 1.81)  *p=0.098* | 1.00 [Reference]  1.26 (0.96, 1.67)  *p=0.094* |
| You had an outstanding personal achievement  (awards, grades, etc.) |  |  |  |  |
| No  Yes | 173/770 (22.5%)  20/87 (23.0%) | 1.00 [Reference]  1.04 (0.70, 1.53)  *p=0.85* | 1.00 [Reference]  1.14 (0.79, 1.65)  *p=0.47* | 1.00 [Reference]  1.03 (0.75, 1.41)  *p=0.88* |
| All analyses by log-binomial regression. ^a^Model 1 adjusted for whether participants were experiencing ongoing symptoms due to recent relapse. ^b^Model 2 further adjusted for age, sex, P-MSSS, and prescription antidepressant medication. ^c^Model 3 further adjusted for age, sex, P-MSSS, prescription antidepressant medication, and clinically significant fatigue.  Results in boldface denote statistical significance (p<0.05).  Abbreviations: PHQ-9 = Patient Health Questionnaire-9; aPR = adjusted prevalence ratio; SLE = stressful life event | | | | |

Supplementary Table 6. Individual SLEs not associated with clinically significant fatigue at 7.5-year review.

| **SLE measure** | **n/N (row %)** | **aPR (95% CI)^a^** | **aPR (95% CI)^b^** | **aPR (95% CI)^c^** |  |
| --- | --- | --- | --- | --- | --- |
| Your parent, child or partner died |  |  |  |  |  |
| No  Yes | 328/807 (40.6%)  24/51 (47.1%) | 1.00 [Reference]  1.12 (0.82, 1.53)  *p=0.47* | 1.00 [Reference]  1.12 (0.83, 1.52)  *p=0.45* | 1.00 [Reference]  1.12 (0.85, 1.47)  *p=0.42* |  |
| Your parent, child or partner died (negative impact only) |  |  |  |  |  |
| No  Yes | 330/809 (40.8%)  22/49 (44.9%) | 1.00 [Reference]  1.07 (0.77, 1.48)  *p=0.70* | 1.00 [Reference]  1.11 (0.80, 1.53)  *p=0.54* | 1.00 [Reference]  1.10 (0.82, 1.46)  *p=0.53*1.00 |  |
| A close family friend or another relative died |  |  |  |  |  |
| No  Yes | 277/690 (40.1%)  75/168 (44.6%) | 1.00 [Reference]  1.10 (0.91, 1.33)  *p=0.34* | 1.00 [Reference]  0.98 (0.81, 1.18)  *p=0.82* | 1.00 [Reference]  1.08 (0.90, 1.29)  *p=0.43* |  |
| A close family friend or another relative died (negative impact only) |  |  |  |  |  |
| No  Yes | 286/708 (40.4%)  66/150 (44.0%) | 1.00 [Reference]  1.07 (0.87, 1.30)  *p=0.53* | 1.00 [Reference]  0.96 (0.79, 1.17)  *p=0.70* | 1.00 [Reference]  1.04 (0.86, 1.26)  *p=0.66* |  |
| You yourself suffered a serious injury or assault |  |  |  |  |  |
| No  Yes | 337/826 (40.8%)  15/32 (46.9%) | 1.00 [Reference]  1.10 (0.77, 1.61)  *p=0.58* | 1.00 [Reference]  1.01 (0.70, 1.48)  *p=0.95* | 1.00 [Reference]  1.10 (0.91, 1.32)  *p=0.31* |  |
| You yourself suffered a serious injury or assault (negative impact only) |  |  |  |  |  |
| No  Yes | 337/829 (40.7%)  15/29 (51.7%) | 1.00 [Reference]  1.22 (0.86, 1.75)  *p=0.27* | 1.00 [Reference]  1.13 (0.79, 1.62)  *p=0.50* | 1.00 [Reference]  1.12 (0.93, 1.35)  *p=0.22* |  |
| A serious illness, injury or assault happened to a close relative or friend |  |  |  |  |  |
| No  Yes | 290/720 (40.3%)  62/138 (44.9%) | 1.00 [Reference]  1.08 (0.88, 1.33)  *p=0.45* | 1.00 [Reference]  1.03 (0.84, 1.25)  *p=0.81* | 1.00 [Reference]  1.04 (0.86, 1.25)  *p=0.72* |  |
| A serious illness, injury or assault happened to a close relative or friend (negative impact only) |  |  |  |  |  |
| No  Yes | 292/731 (40.0%)  60/127 (47.2%) | 1.00 [Reference]  1.14 (0.93, 1.40)  *p=0.20* | 1.00 [Reference]  1.07 (0.88, 1.31)  *p=0.49* | 1.00 [Reference]  1.07 (0.89, 1.30)  *p=0.47* |  |
| You (or your parents) broke off a steady relationship |  |  |  |  |  |
| No  Yes | 339/825 (41.1%)  13/33 (39.4%) | 1.00 [Reference]  0.90 (0.58, 1.41)  *p=0.66* | 1.00 [Reference]  0.91 (0.59, 1.41)  *p=0.68* | 1.00 [Reference]  0.95 (0.63, 1.44)  *p=0.82* |  |
| You (or your parents) broke off a steady relationship (negative impact only) |  |  |  |  |  |
| No  Yes | 341/828 (41.2%)  11/30 (36.7%) | 1.00 [Reference]  0.82 (0.50, 1.35)  *p=0.44* | 1.00 [Reference]  0.83 (0.51, 1.34)  *p=0.44* | 1.00 [Reference]  0.87 (0.55, 1.38)  *p=0.57* |  |
| You became pregnant or menopausal |  |  |  |  |  |
| No  Yes | 316/788 (40.1%)  36/70 (51.4%) | 1.00 [Reference]  **1.29 (1.01, 1.65)**  ***p=0.038*** | 1.00 [Reference]  1.23 (0.95, 1.60)  *p=0.12* | 1.00 [Reference]  1.23 (0.95, 1.59)  *p=0.12* |  |
| You became pregnant or menopausal (negative impact only) |  |  |  |  |  |
| No  Yes | 327/810 (40.4%)  25/48 (52.1%) | 1.00 [Reference]  1.29 (0.97, 1.71)  *p=0.076* | 1.00 [Reference]  1.26 (0.93, 1.71)  *p=0.14* | 1.00 [Reference]  1.23 (0.93, 1.64)  *p=0.15* |  |
| You became engaged married or resumed a steady relationship |  |  |  |  |  |
| No  Yes | 344/838 (41.1%)  8/20 (40.0%) | 1.00 [Reference]  0.99 (0.56, 1.73)  *p=0.97* | 1.00 [Reference]  1.03 (0.63, 1.71)  *p=0.90* | 1.00 [Reference]  0.98 (0.61, 1.59)  *p=0.94* |  |
| You had problems with the police and a court appearance |  |  |  |  |  |
| No  Yes | 348/81 (40.9%)  4/7 (57.1%) | 1.00 [Reference]  1.41 (0.73, 2.73)  *p=0.30* | 1.00 [Reference]  1.43 (0.74, 2.74)  *p=0.28* | 1.00 [Reference]  1.80 (0.99, 3.29)  *p=0.054* |  |
| You were fired/sacked from your job or expelled from school |  |  |  |  |  |
| No  Yes | 351/848 (41.4%)  1/10 (10.0%) | 1.00 [Reference]  0.22 (0.04, 1.33)  *p=0.099* | 1.00 [Reference]  0.27 (0.04, 1.70)  *p=0.16* | 1.00 [Reference]  0.27 (0.05, 1.45)  *p=0.13* |  |
| You were fired/sacked from your job or expelled from school (negative impact only) |  |  |  |  |  |
| No  Yes | 351/849 (41.3%)  1/9 (11.1%) | 1.00 [Reference]  0.24 (0.04, 1.43)  *p=0.12* | 1.00 [Reference]  0.29 (0.04, 1.83)  *p=0.19* | 1.00 [Reference]  0.29 (0.06, 1.53)  *p=0.15* |  |
| You had a crisis or  serious disappointment in your work, school or career |  |  |  |  |  |
| No  Yes | 313/751 (41.7%)  39/107 (36.5%) | 1.00 [Reference]  0.86 (0.66, 1.12)  *p=0.26* | 1.00 [Reference]  0.92 (0.71, 1.19)  *p=0.53* | 1.00 [Reference]  0.88 (0.69, 1.12)  *p=0.30* |  |
| You had a crisis or serious disappointment in your work, school or career (negative impact only) |  |  |  |  |  |
| No  Yes | 315/757 (41.6%)  37/101 (36.6%) | 1.00 [Reference]  0.87 (0.66, 1.14)  *p=0.31* | 1.00 [Reference]  0.92 (0.71, 1.20)  *p=0.55* | 1.00 [Reference]  0.89 (0.69, 1.14)  *p=0.34* |  |
| You gained a new family member (new baby born or parent remarried) |  |  |  |  |  |
| No  Yes | 323/794 (40.7%)  29/64 (45.3%) | 1.00 [Reference]  1.13 (0.85, 1.50)  *p=0.40* | 1.00 [Reference]  1.08 (0.84, 1.39)  *p=0.54* | 1.00 [Reference]  1.10 (0.87, 1.39)  *p=0.43* |  |
| You gained a new family member (new baby born or parent remarried) (negative impact only) |  |  |  |  |  |
| No  Yes | 349/852 (41.0%)  3/6 (50.0%) | 1.00 [Reference]  1.33 (0.59, 2.97)  *p=0.49* | 1.00 [Reference]  1.03 (0.64, 1.68)  *p=0.89* | 1.00 [Reference]  0.87 (0.59, 1.27)  *p=0.47* |  |
| You had a major financial crisis |  |  |  |  |  |
| No  Yes | 324/800 (40.5%)  28/58 (48.3%) | 1.00 [Reference]  1.14 (0.86, 1.51)  *p=0.36* | 1.00 [Reference]  1.05 (0.81, 1.37)  *p=0.71* | 1.00 [Reference]  0.94 (0.74, 1.19)  *p=0.60* |  |
| You had a major financial crisis (negative impact only) |  |  |  |  |  |
| No  Yes | 325/803 (40.5%)  27/55 (49.1%) | 1.00 [Reference]  1.15 (0.87, 1.53)  *p=0.33* | 1.00 [Reference]  1.08 (0.83, 1.40)  *p=0.58* | 1.00 [Reference]  0.96 (0.77, 1.21)  *p=0.76* |  |
| You changed house, school, or jobs |  |  |  |  |  |
| No  Yes | 306/733 (41.8%)  46/125 (36.8%) | 1.00 [Reference]  0.87 (0.68, 1.11)  *p=0.26* | 1.00 [Reference]  0.93 (0.74, 1.18)  *p=0.56* | 1.00 [Reference]  0.86 (0.69, 1.08)  *p=0.20* |  |
| You changed house, school, or jobs (negative impact only) |  |  |  |  |  |
| No  Yes | 328/804 (40.8%)  24/54 (44.4%) | 1.00 [Reference]  1.03 (0.76, 1.40)  *p=0.85* | 1.00 [Reference]  1.09 (0.80, 1.49)  *p=0.57* | 1.00 [Reference]  0.99 (0.75, 1.31)  *p=0.95* |  |
| You changed your personal habits, including use of alcohol or drugs |  |  |  |  |  |
| No  Yes | 333/809 (41.2%)  19/49 (38.8%) | 1.00 [Reference]  0.95 (0.67,1.35)  *p=0.78* | 1.00 [Reference]  0.98 (0.72, 1.34)  *p=0.91* | 1.00 [Reference]  0.89 (0.66, 1.20)  *p=0.46* |  |
| You changed your personal habits, including use of alcohol or drugs (negative impact only) |  |  |  |  |  |
| No  Yes | 345/844 (40.9%)  7/14 (50.0%) | 1.00 [Reference]  1.19 (0.72, 1.96)  *p=0.51* | 1.00 [Reference]  1.27 (0.76, 2.13)  *p=0.36* | 1.00 [Reference]  1.10 (0.68, 1.77)  *p=0.71* |  |
| You had an outstanding personal achievement (awards, grades, etc.) |  |  |  |  |  |
| No  Yes | 318/771 (41.3%)  34/87 (39.1%) | 1.00 [Reference]  0.97 (0.74, 1.27)  *p=0.81* | 1.00 [Reference]  1.14 (0.87, 1.48)  *p=0.34* | 1.00 [Reference]  1.09 (0.86, 1.37)  *p=0.50* |  |
| All analyses by log-binomial regression.  ^a^Model 1 adjusted for whether participants were experiencing ongoing symptoms due to recent relapse. ^b^Model 2 adjusted for model 1 variable and further adjusted for age, sex, P-MSSS, prescription antifatigue medication, and baseline comorbidity number. ^c^Model 3 adjusted for model 2 variables and also adjusted for depression  Results in boldface denote statistical significance (p<0.05).  Abbreviations: aPR = adjusted prevalence ratio; SLE = stressful life event | | | | | |
